# Selective non-operative management for penetrating splenic trauma: a systematic review

**DOI:** 10.1007/s00068-019-01117-1

**Published:** 2019-04-10

**Authors:** Michel Teuben, Roy Spijkerman, Roman Pfeifer, Taco Blokhuis, Josephine Huige, Hans-Christoph Pape, Luke Leenen

**Affiliations:** 1 Department of Trauma, University Medical Center Utrecht, Heidelberglaan 100, 3584 CX Utrecht, The Netherlands; 2 Department of Trauma, University Hospital Zurich, Zurich, Switzerland; 3 Department of Surgery, University Medical Center Maastricht, Maastricht, The Netherlands

**Keywords:** Splenic injury, Penetrating trauma, Selective non-operative management

## Abstract

**Introduction:**

The treatment of abdominal solid organ injuries has shifted towards non-operative management (NOM). However, the feasibility of NOM for penetrating splenic trauma is unclear and outcome is believed to be worse than NOM for penetrating liver and kidney injuries. Hence, the aim of the current systematic review was to evaluate the feasibility of selective NOM in penetrating splenic injury.

**Methods:**

A review of literature was performed using Pubmed, Embase and Cochrane databases. Studies on adult patients treated by NOM for splenic injuries were included and outcome was documented and compared.

**Results:**

Five articles from exclusively level-1 and level-2-traumacenters were selected and a total of 608 cases of penetrating splenic injury were included. Nonoperative management was applied in 123 patients (20.4%, range 17–33%). An overall failure rate of NOM of 18% was calculated. Mortality was not seen in patients selected for nonoperative management. Contra-indicatons for NOM included hemodynamic instability, absence of abdominal CT-scanning to rule out concurrent injuries and peritonitis.

**Conclusions:**

This review demonstrates that non-operative management for penetrating splenic trauma in highly selected patients has been utilized in several well-equipped and experienced trauma centers. NOM of penetrating splenic injury in selected patients is not associated with increased morbidity nor mortality. Data on the less well-equipped and experienced trauma centers are not available. More prospective studies are required to further define exact selection criteria for non-operative management in splenic trauma.

**Electronic supplementary material:**

The online version of this article (10.1007/s00068-019-01117-1) contains supplementary material, which is available to authorized users.

## Introduction

The treatment of blunt splenic injury has shifted towards non-operative management (NOM). Operative intervention remains mandated in the case of peritonitis or hemodynamic instability; however, NOM is the treatment of choice in all other cases [1]. NOM is nowadays attempted in up to 97% of patients with blunt splenic injury. Documented success rates exceed 90% [2–4]. The impetus for the shift towards NOM was the identification of an ‘overwhelming post-splenectomy infection syndrome’(OPSI-syndrome) in asplenic patients [5, 6].

The feasibility of NOM for penetrating splenic injury (PSI) has remained relatively unexplored [7, 8]. Since World War I, routine surgical exploration became standard practice for penetrating abdominal trauma. Later it became clear that not all penetrating abdominal injuries require surgical intervention [8, 9]. In 1960, Shaftan et al. suggested ‘observant and expectant treatment’ as a safe alternative in selected patients [10]. Improvements in diagnostics and patient monitoring led to increased popularity of non-operative approaches for penetrating abdominal injuries [11]. In addition to long-term benefits of preservation of splenic function, negative laparotomies are related with increased complications and mortality rates as well [9, 12]. Feasibility of selective NOM for penetrating abdominal trauma has been demonstrated previously [11]. However, compared with other organs in penetrating blunt abdominal trauma, splenic injury is associated with impaired outcome of NOM [13]. Hence, the aim of the current systematic review was to evaluate the feasibility of selective NOM in penetrating splenic injury.

## Materials and methods

### Research question

To determine the feasibility of selective NOM for penetrating splenic injury, we addressed the following research question: What is the outcome of NOM in adult patients sustaining penetrating splenic injury compared to patients treated by operative management?

Domain: adult patients with penetrating splenic injury.

Determinant: non-operative management.

Primary outcome: mortality rate.

The following endpoints were defined:

*Primary endpoints*:

(1) Mortality rate of patients with penetrating splenic injury treated by NOM.

*Secondary endpoints*:

(1) Failure of NOM; (2) Number and type of complications; (3) Length of intensive care unit (ICU) stay; (4) Length of hospital stay (LOS); (5) Overall mortality rate of all patients (including those treated by OM) treated according to guidelines including nonoperative therapy.

### Data search and search strategy

A systematic review of published literature in the Cochrane, Pubmed and Embase libraries was performed. Preferred Reporting Items for Systematic Reviews and Meta-analyses (PRISMA) recommendations [14] and the ‘Cochrane Collaboration’s tool for assessing risk of bias’ [15] were integrated in our selection procedures.

All articles published within the time period from 1940 till 21st of November 2018 were included. On the 21st of December 2018 we executed a search including domain and determinant of our study. Title and abstract were searched for the terms ‘penetrating splenic injury’ (domain) and ‘non-operative management’ (determinant) and their relevant synonyms and their plural forms. The search query is shown in Supplement 1.

### Study selection

Publications were included in the review if:

(1) A study population including adult (> 16 years) trauma patients with penetrating splenic injury (PSI) was utilized; (2) a study included at least 3 patients treated non-operatively for penetrating splenic injury; (3) a study described primary and secondary outcome (mortality or failure of NOM, complications, length of intensive care unit stay or length of hospital stay); (4) a study is reported in English or German language; (5) a study included original data (no reviews, case-reports, case series, editorial letters, discussions, expert opinions or meeting abstracts). Animal studies were excluded. After removal of duplicates, title and abstract were screened on inclusion and exclusion criteria by three different authors (JH, MT, RS). Subsequently, the full text was analysed. Data extraction was performed as described hereafter and the references were screened.

### Critical appraisal

Standard criteria for assessing therapeutic research were used in our critical appraisal table to assess the relevance and validity of the selected papers. The ‘Cochrane Collaboration’s tool for assessing risk of bias’ is integrated in our selection procedures [15]. The criteria are displayed in Supplement 1. All articles with a cumulative score ≥ 9 or higher were included for data analysis. Discordant judgements were resolved by consensus discussion.

### Data extraction

Data extraction was performed using a standardized checklist for the following characteristics and outcome parameters:

(1) total number of patients with PSI; (2) type of penetrating injury [stab wounds (SW), gunshot wounds (GSW)]; (3) median age; (4) gender-distribution; (5) Injury Severity Score (ISS) [16]; (6) Abbreviated Injury Score (AIS) [17] of splenic injury; (7) number of patients sustaining PSI treated by NOM; (8) number of patients sustaining PSI treated by operative management; (9) failure of NOM; (10) number and type of complications; (11) length of ICU-stay; (12) length of hospital stay (LOS); (13) mortality-rate and absolute risk on mortality.

### Statistical analysis

Patient characteristics and outcome were summarized and pooled using descriptive statistics. Corresponding authors were contacted if the reported data were unclear or incomplete for required data extraction.

## Results

### Search strategy

**Fig. 1:**
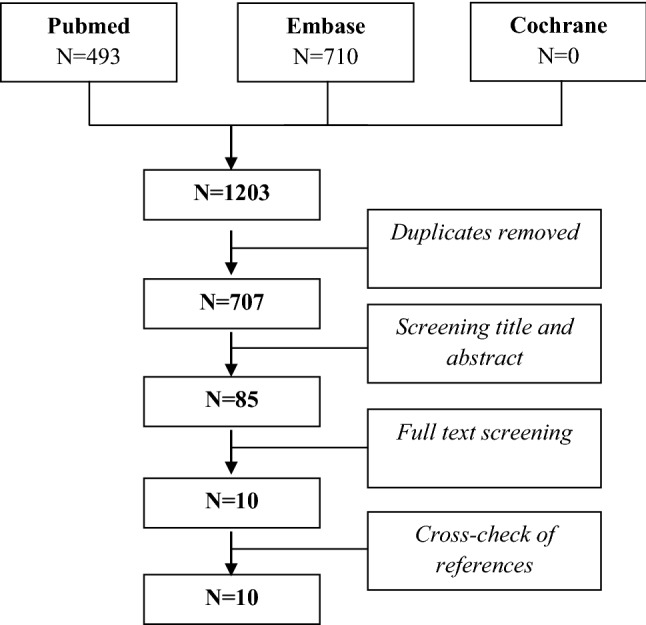
Flowchart

The search yielded a total of 1203 publications, of which 707 were unique. After screening for title and abstract, 85 articles were selected for full-text screening. After full-text screening 10 articles were included in the critical appraisal procedure [18–27]. References of these articles were screened for additional relevant studies. No extra articles were retrieved in this manner (Fig. 1).

In the critical appraisal procedure six articles scored > 8 points (Supplement 2). The studies published by Berg et al. in 2014 [25] and Demetraides et al. [21] were performed in the same institute. Furthermore, time periods of both studies were partly overlapping each other. Therefore, the latter study was excluded. After these steps a total of five studies (one prospective and four retrospective studies) were utilized for data extraction [19, 20, 23, 25, 27].

### Study populations

The largest study was a single-center study (level one trauma center) performed by Berg et al. [25]. They retrospectively included 225 patients with penetrating splenic trauma. The second largest study was a retrospective dual center study performed by Spijkerman et al. [27]. For this study, a total of 118 patients were included in the participating level one trauma centers. A retrospective study conducted by Clancy et al. [19], they investigated outcome of penetrating splenic injury in 197 patients admitted to level I/II trauma centers. Patients were grouped based on age. The first group consisted of 188 adult patients, group two included 9 geriatric patients. Pachter et al. [20] reported in their experience with selective NOM in patients treated in a level one trauma center. Forty-three patients with penetrating splenic injuries were prospectively included. In a retrospective descriptive study executed by Kaseje et al. [23] a total of 25 patients with penetrating splenic injury were included from an urban level one trauma database. Patient and trauma characteristics of all populations are summarized in Table 1.

**Table 1:** Patient and injury characteristics

References	No. of pts with PSI	Patient characteristics	Injury characteristics	Attempted NOM
Clancy et al. [19]	188	Age: 17–64	Mean ISS (std): 21.8 (± 9.7)	54 (29%)
Clancy et al. [19]	9	Age: > 64	Mean ISS (std): 21.4 (± 11.2)	3 (33%)
Pachter et al. [20]	43	n/a	18 GSW and 25 SW	6 (14%); only stab wounds
Kaseje et al. [23]	25	Mean age: 28.6, 23M/2 F	Mean ISS: 19.6	5 (20%)
Berg et al. [25]	225	Mean age: 28, 217M/8 F	146 GSW ISS > 24: 40%	38 (17%)
Spijkerman et al. [27]	118	Mean age 27, 109M/9 F	Median (IQR) ISS: 25 65 GSW and 53 SW	22 (19%)

*Pts* patients, *std* standard deviation, *PSI* penetrating splenic injury, *n/a* not available, *M* male, *F* female, *ISS* Injury Severity Score, *GSW* gunshot wounds, *SW* stab wounds

### Management and outcome

Thirty-eight out of 225 patients with splenic trauma studied by Berg et al. were selected for NOM. Of these thirty-eight NOM patients, 14 patients failed NOM and required emergency surgery. Mortality was not seen in patients selected for NOM. The NOM patients had significantly better hemodynamics (3% vs. 28% hypotension on admission (*p* = 0.024). Furthermore, NOM-group patients were less often injured through a gunshot injury (34% vs. 71%, *p* < 0.001), included less patients with high-grade splenic injuries (40% vs. 62%, *p* = 0.018) and had less patient with an ISS > 24 (13% vs. 46%, *p* < 0.001) compared with patients selected for operative therapy. In addition, patients who underwent early operative treatment were more frequently splenectomized (55% vs. 8%), had significantly higher mortality rates (17% vs. 0%), longer median ICU stays (3 vs. 0 days) and longer hospital-LOS (9 vs. 4 days) compared with those patients selected for NOM [25].

In the study from Spijkerman et al. 45 patients were splenectomized, 51 patients were treated with a spleen-preserving operative intervention and 22 patients were treated by NOM (18.6%). The median (IQR, interquartile range) ICU-stay was shorter in NOM patients compared with splenectomized patients [0 (0–1) vs. 2 (0–6) days]. However, median (IQR) hospitalization times were comparable between NOM patients [8 (5–15) days] and splenectomized cases [8 (7–12) days]. Additionally, no differences were seen in the frequency of complications between groups. Moreover, mortality was not seen in patients treated by NOM [27].

Clancy et al. studied differences between adult and geriatric trauma cases. They included 54 (out of a total of 188) adult patients with splenic trauma (< 65 years) that were selected for non-operative management. They did not document failure rates of NOM nor complications. The overall mortality rate, including both patients treated through NOM and through OM, was 8.6%. Additionally, out of nine geriatric patients, three patients were selected for NOM. Four out of 9 patients deceased. After assembling outcome data of both study groups, a total mortality rate of 20/197 (10.2%) was found. Non-operative management was attempted in 57 out of 197 patients [19].

In the study described by Pachter et al., six patients with stab wounds were selected for NOM. None of them failed NOM and all patients recovered uneventfully [20]. Kaseje et al. included five cases of NOM. All the patients selected for NOM had an uncomplicated clinical course and failure of conservative management did not occur. The mean length of hospital stay for all 25 patients sustaining penetrating splenic injury was 13.5 days and length of ICU-stay was 6.6 days [23]. Table 2 shows outcome data.

**Table 2:** Outcome of non-operative management

References	No. of patients	Attempted NOM	Outcome
Clancy et al. [19]	188	54	Mean hospital LOS (std): 18.6 (29.6) days^a^ Total mortality: 8.6%^a^
Clancy et al. [19]	9	3	Mean hospital LOS (std): 28.3 (35.1) days^a^ Total mortality: 44%^a^
Pachter et al. [20]	43	6	Failure rate iNOM: 0/6 No. of complications: 0 Mortality iNOM: 0%
Kaseje et al. [21]	25	5	Failure rate iNOM: 0/5 No. of complications: 0 Mean hospital LOS (std): 13.5 (1–42) days Mortality iNOM: 0%
Berg et al. [25]	225	38	Failure rate iNOM: 9 (24%) No. of complications: 3 No. of patients with complications: 2 Mean ICU-stay: 0 (range 0–10) days Mean hospital LOS (range): 4 (1–19) days Mortality iNOM: 0% Total mortality: 14%
Spijkerman et al. [27]	118	22	Failure rate iNOM: n/a No. of complications: 5 No. of patients with complications: 4 Mean ICU-stay: 0 (IQR, 0–1) days Mean hospital LOS: 8 (IQR, 5–15) days Mortality iNOM: 0% Total mortality: 5.9%

*n/a* not available, *std* standard deviation, *No* number, *iNOM* initially attempted non-operative management, *LOS* length of stay, *IQR* interquartile range

^a^Overall outcome (including both NOM and OM patients)

### Pooling of data

In all selected studies, a total of 608 patients sustained penetrating splenic injuries. Non-operative management was applied in 123 patients (20.4%, range 17–33%). A cumulative failure rate of initial NOM of 18% (range 0–24%) was calculated. Mean LOS (range) in patients treated with selective NOM ranged from 4 (range 1–19) days in the study performed by Berg et al. to 28.3 (35.1) in the geriatric patients from Clancy’s study [19, 25]. In 90% (range 82–100%) of successful NOM the clinical recovery was uneventful. A total pooled mortality rate of 11% was seen in patients treated for penetrating splenic trauma, whereas mortality in 123 patients treated through NOM was not observed.

## Discussion

This review is the first to determine current evidence in literature for the feasibility of selected non-operative management in penetrating splenic injury. This study demonstrates that:

1. Non-operative management for penetrating splenic trauma in highly selected patients has been utilized in several well-equipped and experienced trauma centers.
2. NOM of penetrating splenic injury in selected patients is not associated with increased morbidity nor mortality.
3. Data on the safety and feasibility of NOM for penetrating splenic trauma in less well-equipped and experienced trauma centers are not available yet.

The feasibility of NOM in penetrating splenic injury is relatively unexplored. Our extensive literature search identified five articles and it became clear that selective NOM has been implemented and utilized in some high-volume institutions. An overall mortality rate in patients treated (both operatively and nonoperatively) for penetrating splenic trauma of 11% was observed. This is comparable to studies were NOM is not utilized as treatment modality for penetrating splenic trauma [28].

A total of 123 patients were treated by NOM. A trial of NOM in patients was found not to be associated with increased morbidity nor mortality. Therefore, we believe that in well-equipped and experienced trauma centers a trial of NOM is a feasible treatment option for penetrating splenic trauma in selected patients. This is in line with findings from reviews on selective nonoperative therapy for other solid organ injuries [11].

It is important to realize that we did not find any data on low-volume institutes. In our opinion penetrating splenic injuries are treated best by surgical exploration in low-volume centers. In our opinion more studies are required to further evaluate the feasibility of NOM for splenic trauma under these specific conditions. Furthermore, patients with splenic GSWs were not studied in detail and tend to have impaired outcome. So, in our view selection criteria in these patients should be even more strict and monitoring conditions should be optimal.

Adequate patient selection is a prerequisite for successful non-operative therapy. When comparing treatment guidelines and selection criteria between studies we encountered several differences. Utilized exclusion criteria for a trial of NOM are summarized in Supplement 3. Despite minor differences, Berg et al. [25] and Spijkerman et al. [27] utilized comparable selection criteria for non-operative therapy. Patients analyzed by Berg et al. [25] with either clinical signs of peritonitis, hemodynamic instability or those patients unable to respond to clinical examination were selected for laparotomy. Patients without hemodynamic abnormalities underwent CT-scanning to identify concomitant intra-abdominal lesions. Patients without relevant intra-abdominal injuries requiring surgical intervention (such as hollow organ injuries, pancreatic injuries) were selected for NOM. Those with left-sided thoracoabdominal trauma were scheduled for a diagnostic laparoscopy in order to determine occult diaphragmatic injuries [25]. Spijkerman et al. suggest NOM in patients without hollow viscus injuries, hemodynamic instability, decreased level of consciousness, spinal cord injuries, blood in nasogastric tube and blood on rectal examination. All patients selected for NOM underwent CT-scanning to rule out concurrent injuries [27]. In the study conducted by Kaseje et al. [23] a total of five patients were successfully treated with NOM in an urban level one trauma centre, but no strict treatment guidelines were documented. The choice of treatment was made by the attending trauma surgeon and all conservatively treated patients had relatively minor splenic injuries without signs of ongoing blood loss. Clancy et al. [19] selected patients admitted between January 1988 and December 1993. Hence, criteria and outcome in this study might be slightly outdated. Factors affecting the decision-making process, as well as treatment guidelines were not documented in their publication [19]. Pachter et al. [20] showed promising results after selective NOM in 43 patients with penetrating splenic injuries. They reviewed all patients presented between 1990 and 1996 with splenic injuries. As this study was performed more than 20 years ago treatment guidelines might have changed afterwards. According to their algorithm, all patients with gunshot wounds underwent immediate celiotomy. In stab-wound injuries, management was based on hemodynamic status. Hemodynamically stable patients were considered as candidates for conservative therapy. Patients with anterior stab wounds underwent tractotomy under local anesthesia to determine the presence of peritoneal penetration. In the presence of peritoneal perforation, a celiotomy was performed. If the patient was stabbed in the back or in the side, CT scanning was performed. Patients with isolated splenic injury without evidence of further hemorrhage were selected for NOM. Further contra-indications for NOM were the presence of surgery requiring concurrent intra-abdominal injuries detected on CT scan and more than 2 units transfusion of blood products related to the splenic injury [20]. Interestingly, most articles did not mention the utilization of a laparoscopy in the evaluation and treatment of penetrating splenic trauma—except for the study performed by Berg et al., in which a laparoscopy is performed to evaluate potential diaphragmatic injuries. Laparoscopy and peritoneal lavage were not mentioned in the included studies as a diagnostic or therapeutic tool for penetrating splenic trauma. In our view, upcoming studies should focus on the feasibility of laparoscopy to evaluate and treat splenic penetrating trauma as well.

Nowadays selective non-operative management of renal and liver trauma is recommended in patients without hemodynamic instability or signs of hollow organ injuries [11]. The feasibility of NOM in the treatment of splenic injuries has not been reviewed in detail previously and patients with penetrating splenic trauma are at higher risks of NOM failure than patients with renal or hepatic injuries [13]. The benefits of NOM and preservation of splenic function should be considered carefully when comparing to the risks of missed abdominal concurrent injuries and increased blood loss from the injured spleen.

To minimize missed injuries and a delayed intervention, mandatory celiotomy is still the treatment of choice for PSI in most institutions. However, this procedure showed to be unnecessary in 23–53% of patients with abdominal stab wounds. Furthermore, negative laparotomy in trauma patients has a complication rate of 2.5–41% and unnecessary celiotomy is related to increased mortality [9, 12]. Moreover, laparotomy can lead to long-term complications such as hollow viscus obstruction and incisional hernias [29]. Spijkerman et al. encountered 7 complications in 22 patients treated by NOM. Two intra-abdominal abscesses were encountered, and two patients developed pneumonia. No hollow organ injuries were missed in the study from Spijkerman et al. [27], and in patients selected for NOM by Berg et al. [25]. The other included studies did not describe complications in patients treated by NOM in detail. Therefore, the amount of missed injuries in included non-operatively treated patients is unclear. This is the main limitation of our study.

In conclusion, our study indicates that a trial of NOM in highly selected patients is not associated with increased morbidity nor mortality in high-volume trauma centers. Therefore, we suggest that a trial of NOM for penetrating splenic injury can be safely applied in selected patients. Prerequisites for successful NOM include hemodynamical stability, no signs of peritonitis, a CT-scan without signs of hollow viscus injury or diaphragm injuries. Relative contra-indications for NOM included impaired mental status and spinal injuries, blood in nasogastric tube or blood on rectal examination as well as high (> 2 units of red blood cells) spleen-related transfusion requirements. Furthermore, adequate continuous hemodynamic monitoring should be available, and serial physical examinations as well as laboratory tests (serum haemoglobin) should be performed.

We further suggest operative intervention for penetrating splenic trauma in low-volume centers, rather than a trial of NOM, as the external validity of the presented data for these centers is unclear. Moreover, outcome of NOM for GSWs seems to be impaired and therefore selection protocols in these patients should be followed even more strictly. As guidelines differ between institutions, more prospective studies are required to further define selection criteria for NOM in penetrating splenic trauma.

## Electronic supplementary material

Below is the link to the electronic supplementary material.

Supplementary material 1 (DOCX 19 kb)
