# Supplementary material for: Selective non-operative management for penetrating splenic trauma: a systematic review
Source: Eur J Trauma Emerg Surg. 2019 Apr 10;45(6):979–85. doi: 10.1007/s00068-019-01117-1 (PMC6910899; doi:10.1007/s00068-019-01117-1)
Supplement: Supplementary file 1 — Supplementary material 1 (DOCX 19 kb) [file 68_2019_1117_MOESM1_ESM.docx]

Supplement 1: **search query (Pubmed and adapted EMBASE version)**

(penetrate OR penetrating OR perforate OR perforating OR ballistic OR ballistics OR gunshot OR gunshots OR ‘gun shot’ OR ‘gun shots’ OR shotgun OR shotguns OR ‘shot gun’ OR ‘shot guns’ OR stabwound OR stabwounds OR stab OR stabs OR stabbing OR knife) AND (abdomen OR abdominal OR intraabdominal OR ‘intra abdominal’ OR ‘intra-abdominal’ OR thoracoabdominal OR ‘thoraco abdominal’ OR ‘thoraco-abdominal’ OR intraperitoneal OR ‘intra peritoneal’ OR ‘intra-peritoneal’ OR flank OR spleen OR splenic) AND (injury OR injuries OR injured OR trauma OR rupture OR ruptured OR ruptures OR lesion OR lesions OR laceration OR lacerations OR damage OR damaged OR tear OR tears OR wound OR wounds) AND (non-operative OR non-operatively OR ‘non operative’ OR ‘non operatively’ OR ‘non-operative’ OR ‘non-operatively’ OR nonsurgical OR nonsurgically OR ‘non surgical’ OR ‘non surgically’ OR ‘non-surgical’ OR ‘non-surgically’ OR conservative OR conservatively OR observational OR observative OR observationally OR ‘selective management’)

Supplement 2: **Critical appraisal**

| **Study** | **Spijkerman et al. [27]** | **Navsaria et al.[26]** | **Berg et al.[25]** | **Fikry et al.[24]** | **Kaseje et al.[23]** | **Dubose et al.[22]** | **Demetriades et al.[21]** | **Pachter et al.[20]** | **Clancy et al.[19]** | **Leppaniemie et al.[18]** |
| --- | --- | --- | --- | --- | --- | --- | --- | --- | --- | --- |
| Year | 2017 | 2015 | 2014 | 2011 | 2008 | 2007 | 2006 | 1998 | 1997 | 1996 |
| Study design | RS | PS | RS | RS | RS | RS | PS | PS | RS | PS |
| No. of patients | 118 | 1106 | 225 | 150 | 170 | 644 | 152 | 109 | 1255 | 51 |
| **Relevance** |  |  |  |  |  |  |  |  |  |  |
| Domain (2p) | + | + | + | + | + | + | + | + | + | + |
| Determinant (2p) | + | + | + | - | + | + | + | + | + | - |
| Outcome (2p) | + | + | + | - | + | - | + | + | + | - |
| *Subtotal* | *6* | *6* | *6* | *2* | *6* | *4* | *6* | *6* | *6* | *2* |
| **Validity**  *(Selection bias)* |  |  |  |  |  |  |  |  |  |  |
| Adjustment for confounding (1p) | - | - | - | - | - | - | - | - | - | - |
| Randomisation (RCT) (1p) | - | - | - | - | - | - | - | - | - | + |
| Concealed allocation (RCT) (1p) | - | - | - | - | - | - | - | - | - | - |
| Baseline (1p) | - | - | - | - | - | - | - | - | - | - |
| Length of follow-up (1p) | + | + | + | - | + | + | + | + | + | + |
| Loss to follow up (1p) | + | - | + | + | + | + | + | + | + | + |
| Missing data (1p) | + | - | + | + | + | + | + | + | - | + |
| *Subtotal* | *2* | *1* | *3* | *2* | *3* | *3* | *3* | *3* | *3* | *4* |
| **Validity** *(Information bias)* |  |  |  |  |  |  |  |  |  |  |
| Standardisation (1p) | + | + | + | + | + | + | + | + | + | + |
| Blinding (1p) | - | - | - | - | - | - | - | - | - | + |
| *Subtotal* | *1* | *1* | *1* | *1* | *1* | *1* | *1* | *1* | *1* | *2* |
|  |  |  |  |  |  |  |  |  |  |  |
| **Total score** | **10** | **8** | **10** | **5** | **10** | **8** | **10** | **10** | **9** | **8** |
| **Selected ( >9)** | **Yes** | **No** | **Yes** | **No** | **Yes** | **No** | **Yes** | **Yes** | **Yes** | **No** |

*Abbreviations:RS; retrospective, PS; prospective.*

| **Relevance:** *Domain:*  + Study included adult patients with diagnosed penetrating splenic injuries - Study included other patients *Determinant:*  + Patients treated by nonoperative management are described - No patients treated by nonoperative management are not described *Outcome*:  + Mortality is mentioned - Mortality is not mentioned  **Validity:** *Adjustment for confounding* + Adjustment for confounders is done - No adjustment for confounding or not mentioned *Randomization* + Groups are properly randomized - Groups are not randomized or randomization is not mentioned *Concealed allocation* + Concealed allocation - No concealed allocation or concealed allocation is not mentioned *Intention to treat analysis* + Intention to treat analysis  - Per protocol analysis or way of analysis is not mentioned *Equal baseline characteristics* + Equal baseline characteristics in all groups - Differences in baseline characteristics or baseline characteristics are not mentioned *Length of follow-up* + Duration of follow up is mentioned - Duration of follow up is mentioned *Loss to follow-up* + < 10% loss to follow up - > 10% loss to follow up or loss to follow up is not mentioned *Missing data* + <10% and equally distributed over groups - >10% or not equally distributed over groups or distribution of missing data is not mentioned *Standardization*  + standardization in diagnosis and assessment - no standardization in diagnosis and assessment or standardization is not mentioned *Blinding* + Single/double or triple blinded - No blinding or blinding procedure is not mentioned |
| --- |

Supplement 3: **Overview of exclusion criteria for a trial of nonoperative management in penetrating splenic injury**

| **Criteria** | **Pachter et al. [20]** | **Berg et al. [25]** | **Spijkerman et al. [27]** |
| --- | --- | --- | --- |
| **Gunshot wound** | X | No exclusion | No exclusion |
| **Hemodynamic instability** | X | X | X |
| **Spleen related transfusion >2 packed RBCs** | X | n/a | n/a |
| **Peritonitis** | X | X | X |
| **Blood on rectal examination** | n/a | n/a | X |
| **Blood in nasogastric tube** | n/a | n/a | X |
| **No CT-abdomen to confirm isolated splenic trauma** | X | X | X |
| **High grade splenic injury** | No exclusion | No exclusion | No exclusion |
| **Haematoperitoneum ≥ 250ml** | No exclusion | n/a | n/a |
| **Impaired mental status** | No exclusion | n/a | X |
| **Underlying disease*** | No exclusion | n/a | n/a |
| **Extra abdominal surgery mandated** | No exclusion | No exclusion | No exclusion |
| **Diagnostic laparoscopy** | n/a | ** | n/a |

*Studies from Clancy et al. [19] and Kaseje et al. [23] were excluded as no data on selection criteria was documented. Abbreviations: X; exclusion criteria. n/a; not available, RBC; red blood cell *; Co-morbidities; HIV, Sickle-cell disease, infectious mononucleosis, leukemia. **; Performed in left thoraco-abdominal trauma to rule out diaphragm injury.*
